# Adult Human Vascular Smooth Muscle Cells on 3D Silk Fibroin Nonwovens Release Exosomes Enriched in Angiogenic and Growth-Promoting Factors

**DOI:** 10.3390/polym14040697

**Published:** 2022-02-11

**Authors:** Peng Hu, Anna Chiarini, Jun Wu, Zairong Wei, Ubaldo Armato, Ilaria Dal Prà

**Affiliations:** 1 Human Histology & Embryology Section, Department of Surgery, Dentistry, Paediatrics & Gynaecology, University of Verona Medical School, 37134 Verona, Italy; peng.hu@univr.it (P.H.); uarmato@gmail.com (U.A.); 2 Department of Burns & Plastic Surgery, The Affiliated Hospital of Zunyi Medical University, Zunyi 563000, China; zairongwei@sina.com; 3 Department of Burns and Plastic Surgery, Second People’s Hospital, University of Shenzhen, Shenzhen 518000, China; junwupro@126.com

**Keywords:** silk fibroin, nonwovens, human, smooth muscle cells, vascular endothelial cells, exosomes, cytokines, chemokines, proliferation, mobilization, angiogenesis

## Abstract

Background. Our earlier works showed the quick vascularization of mouse skin grafted *Bombyx mori* 3D silk fibroin nonwoven scaffolds (3D-SFnws) and the release of exosomes enriched in angiogenic/growth factors (AGFs) from in vitro 3D-SFnws-stuck human dermal fibroblasts (HDFs). Here, we explored whether coronary artery adult human smooth muscle cells (AHSMCs) also release AGFs-enriched exosomes when cultured on 3D-SFnws in vitro. Methods. Media with exosome-depleted FBS served for AHSMCs and human endothelial cells (HECs) cultures on 3D-SFnws or polystyrene. Biochemical methods and double-antibody arrays assessed cell growth, metabolism, and intracellular TGF-β and NF-κB signalling pathways activation. AGFs conveyed by CD9^+^/CD81^+^ exosomes released from AHSMCs were double-antibody array analysed and their angiogenic power evaluated on HECs in vitro. Results. AHSMCs grew and consumed *D*-glucose more intensely and showed a stronger phosphorylation/activation of TAK-1, SMAD-1/-2/-4/-5, ATF-2, c-JUN, ATM, CREB, and an IκBα phosphorylation/inactivation on SFnws vs. polystyrene, consistent overall with a proliferative/secretory phenotype. SFnws-stuck AHSMCs also released exosomes richer in IL-1α/-2/-4/-6/-8; bFGF; GM-CSF; and GRO-α/-β/-γ, which strongly stimulated HECs’ growth, migration, and tubes/nodes assembly in vitro. Conclusions. Altogether, the intensified AGFs exosomal release from 3D-SFnws-attached AHSMCs and HDFs could advance grafts’ colonization, vascularization, and take in vivo—noteworthy assets for prospective clinical applications.

## 1. Introduction

Various insect and arthropod species synthesize complex structural proteins, generically named fibroins [1]. Repeated sequences of three amino acids, Gly-Ser-Gly and Ala-Gly-Ala, denote the biochemical structure of purified silk fibroin (SF) from domesticated *Bombyx mori* silkworm [2]. Macromolecular SF occurs in soluble α-helix or random coil and in insoluble β-sheet forms [3]. Degummed (i.e., sericin-deprived) SF microfibers from silkworms’ cocoons are suitable for producing textiles, surgical sutures, and biomaterial scaffolds. Conversely, random coil or α-helix SF forms are not apt for mechanically adequate scaffolds [3]. Notably, SF’s intrinsic plasticity and many available SF processing methods have allowed the design of versatile scaffolds proper for biomedical tissue engineering/regeneration applications [4]. In fact, native SF microfibers in β-sheet form enjoy good biomechanical properties, biocompatibility, and biodegradability, while lacking significant cytotoxicity and immunogenicity. In addition, various other β-sheet SF forms, e.g., films, sheets, electrospun mats, hydrogels, and nanofibers, were considered as prospective therapeutic tools to engineer, for instance, skin, cartilage, and corneal tissues [5,6,7,8]. Remarkably, the success of biomaterial implants crucially depends, among other factors, on the host’s reaction in terms of neovascularization, regenerating tissue organization, and immune/foreign body responses [9]. In relation to translational medicine, another hidden advantage proper of three-dimensional (3D) SF scaffolds is that humans have well over 50 proteins carrying significant stretches of conserved amino acid sequences also present in *Bombyx mori*’s SF. This evolutionary relationship is at the root of the remarkable biocompatibility and lack of immunogenicity of the SF scaffolds [10,11,12]. Due to these promising features, various SF-based scaffolds/devices have undergone preclinical testing in vitro and in animal models in vivo, some becoming objects of clinical trials, and even entering the market in rare cases [13].

SF scaffolds structured as 3D nonwovens (SFnws) [14] have been our research focus in the skin engineering/regeneration field of endeavour [10,11,15]. Initially, we made two types of SFnws, either by gluing their randomly oriented microfibers with formic acid (FA) or by tangling them via textile carding/needling (C/N) technology [10,11,15]. Once grafted into the subcutaneous tissue of C57/BL6 mice, both FA- and C/N-SFnws guided the successful engineering of a reticular connective tissue integrating the SF microfibers in three-to-six months’ time lags [10,11]. Remarkably, by one month after grafting, abundant proliferating capillaries already grew, first along the SF microfibers and next into the intervening voids in close association with fibroblasts and a few macrophages, multinucleated giant cells, and leukocytes. The upshot was a vascularized tissue that lacked any sign of inflammation, foreign body response, fibrosis, or peripheral encapsulation. The biological mechanism(s) underlying the in vivo intense neovascularization of the FA- and C/N-3D-SFnws remained undetermined. However, both 3D-SFnws showed biomechanical shortcomings such as stiffness and fragility [16]. To address the latter, we combined the carding (C) and hydroentangling (HE) textile technologies to produce a third biomechanically more satisfactory SFnws. On such C/HE SFnws we cultured human dermal fibroblasts (HDFs) in vitro to assess whether they would release exosomes carrying any amounts of angiogenic/growth factors (AGFs) [16]. Reports existed that exosomes released from mesenchymal stem cells (MSCs) and endothelial cells (ECs) promoted vascular endothelial cells (ECs) regeneration and angiogenesis [17,18].

Exosomes are nanoscale (30–120 nm in diameter) membranous extracellular vesicles originating in cells’ multivesicular bodies [19,20,21]. They neatly differ from apoptotic bodies [22]. Once released extracellularly, exosomes transport variable combinations of proteins, lipids, DNA, and RNAs [23,24] they shelter from any environmental breakdown mechanism. Thus, exosomes do travel through the extracellular matrix (ECM) and body fluids (blood, cerebrospinal fluid, urine, and saliva) to reach nearby or far away target cells to which they hand over their complex cargoes via interactions with plasma membranes surface receptors or after endocytosis [19]. The various exosome-conveyed agents affect intracellular signalling pathways; homeostatic mechanisms; antigen presentations; inflammatory processes; blood clotting; cell growth, migration, and death; and angiogenesis/vascularization [16,25,26,27]. Indeed, the exosomes released from the C/HE SFnws-grown HDFs carried heightened amounts of twelve AGFs and potently induced cultured human ECs (HECs) to quickly form abundant endothelial tubes in vitro [16]. One of the queries the latter results raised was whether, besides HDFs, other cell types adhering to C/HE-3D-SFnws-based scaffolds might also release exosomes carrying enriched sets of AGFs.

The smooth muscle cells (SMCs) are relevant to this query as they abound in the layers (*tunicae*) of hollow viscera, vessels included. SMCs embryological origins are multiple, i.e., in the mesoderm’s lateral plate (viscera); in its proepicardium derivative (coronary arteries); and in the neural crest (ascending aorta, aortic arch, and pulmonary trunk) [28,29,30,31,32]. SMCs also derive from ECs’ transdifferentiation [33]. Typically, SMCs phenotypic plasticity is striking, as it ranges with intermediate graduations from the quiescent/contractile to the proliferating/secretory/migratory one [34,35]. This highly modulable phenotypic diversity is crucially relevant to pathological neointima formation and vascular remodelling [36]. Interestingly, mesoderm lateral plate-derived vascular SMCs supported the survival of complex ECs networks in 3D Matrigel cocultures in vitro [37].

Variously structured SF scaffolds modelled as arterial vessels have supplied promising preclinical results [38,39]. Hence, it seemed worth investigating the interactions between human coronary artery SMCs and C/HE-3D-SFnws in view of prospective applications as artificial bypass grafts or devices for cardiac revascularization [32]. However, as the proepicardium, from which coronary artery SMCs stem, derives from the lateral plate mesoderm [32], we hypothesized that our study might throw light on the behaviour of SMCs inhabiting hollow viscera walls (e.g., airways, intestine, or bladder), which too stem from the mesodermal lateral plate [40,41]. Moreover, SMCs produce and release compounds such as soluble enzymes, growth factors, cytokines, and chemokines, which in their turn affect cell growth and/or differentiation and/or apoptosis, innate immunity, inflammation, angiogenesis, and cancer onset and metastasis [42,43,44,45,46,47]. Vascular SMCs, too, release exosomes, carrying variable loads of proteins; those so far identified were related to focal adhesion and ECM constituents [48]. 

Therefore, in this work, we investigated both the activation of growth-relevant intracellular signalling pathways in nontumorigenic adult human SMCs (AHSMCs) cultured on C/HE-3D-SFnws in vitro and their concurrent release of AGFs by way of exosomes, using as comparison terms AHSMCs grown on polystyrene. We report here that various constituents of TGF-β and NF-κB intracellular signalling pathways were phosphorylated and hence activated more intensely in the C/HE-3D-SFnws-stuck AHSMCs than in polystyrene-adhering ones. We also show that the former cells discharged exosomes carrying 43 different AGFs, eight of which in significantly richer amounts than the latter. Moreover, we prove that the AGFs conveyed by exosomes released from C/HE-SFnws-attached AHSMCs powerfully stimulate human microvascular endothelial cells (HECs) to proliferate, migrate, and form tubes and nodes in vitro. 

## 2. Materials and Methods

### 2.1. C/HE-3D-SFnws

**Figure 1:**
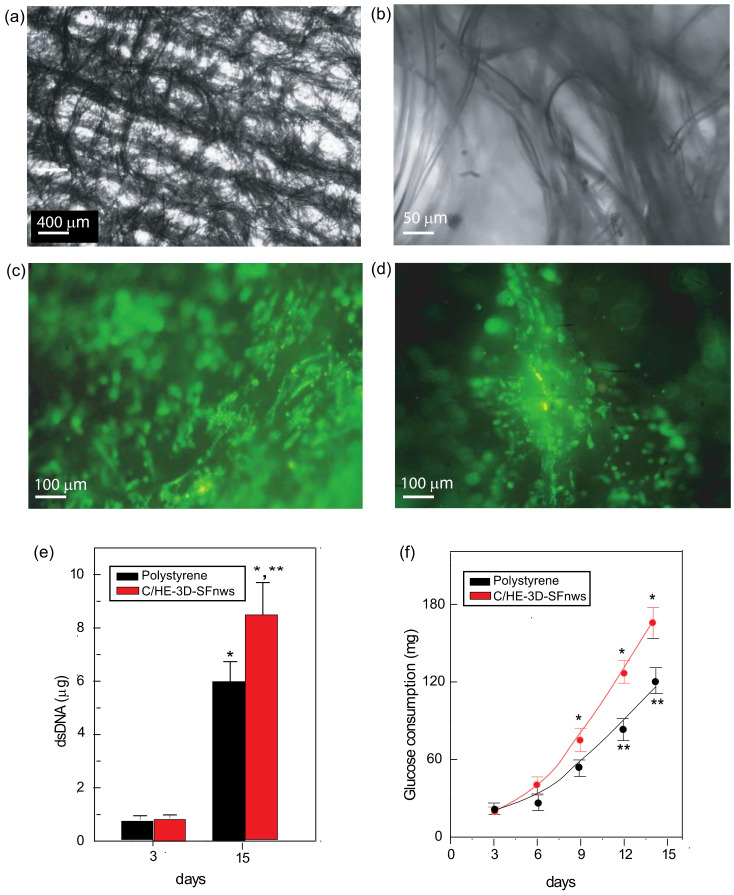
Three-dimensional SF nonwovens (C/HE-3D-SFnws) produced via the carding/hydroentanglement technology are scaffolds supporting the growth of AHSMCs. (**a**,**b**) In these cell-free C/HE-3D-SFnws fabrics the *SF* microfibers form thick longitudinal bundles along the carding machine direction joined by transversal bridges created by the hydroentanglement technology. Light microscope; at low (50×; **a**), and high (500×; **b**) magnifications. (**c**,**d**) Five days after seeding intravitally prestained AHSMCs have adhered along an SF microfiber bundle and have also started colonizing the nearby intercalated voids. (**e**) AHSMCs attached to 3D-SFnws intensely grew between 3 and 15 days of staying in vitro as shown by their 10.9-fold increase in number, while the polystyrene-stuck AHSMCs rose by 7.8-fold. Double strand (ds)DNA amounts were assayed as detailed in the Section 2.5 Bars are mean values ± standard deviations (SDs) from three distinct duplicate determinations at each time point. *, *p* < 0.05 vs. day 3; **, *p* < 0.05 between the two groups at day 15. (**f**) The cumulative *D*-glucose consumption of AHSMCs. Between day 3 and 15, its uptake from the growth medium on the part of C/HE-3D-SFnws-bound AHSMCs increased by 8.4-fold vs. the 6.1-fold of polystyrene-attached AHSMCs. *D*-glucose levels of the AHSMC-conditioned media at each time point were assayed as detailed in the Section 2.6. Each dot stands for the mean value ± SDs from three distinct duplicate determinations. *, *p* < 0.05, vs. day 3 values; **, *p* < 0.05 between the time-corresponding values of the two groups.

Comber waste-derived sericin-deprived (via standard degumming) spun silk in staple form (average fibres length, 50 ± 7 mm) was used to produce the C/HE-3D-SFnws. A cotton type flat carding machine (width, 100 cm) first processed this SF material. Carding arranged the fibres in bundles preferentially oriented lengthwise, i.e., aligned in the longitudinal direction of the nonwoven web. Next, a mechanical hydroentanglement created numerous bonding points on both surfaces of the carded web (Figure 1a,b) [16].

### 2.2. C/HE-3D-SFnws Samples for In Vitro Cell Cultures

After thorough soaking in PBS, C/HE-3D-SFnws samples underwent transversal cutting into 66 × 23 mm rectangular pieces. The latter were sterilized at 55 °C in a vacuum oven for 3 h through exposure to an ethylene oxide/CO_2_ (10/90 *v*/*v*) mixture under a pressure of 42 psi. Next, the specimens were kept for 24 h in an aeration room followed by an 8 h degassing at 50 °C in a vacuum oven. Before use, the sterilized 3D-SFnws pieces were checked for the absence of morphological changes and the upkeep of mechanical properties. Finally, the sterilized C/HE-3D-SFnws samples were aseptically transferred to 4-well, multi-dish w/lid sterile, polystyrene culture plates (Cat. No. 176597, Nalge Nunc International, Rochester, NY, USA). Sterilized steel rods kept the 3D-SFnws samples edges at the bottom of the plates. 

### 2.3. Cells

Nontumorigenic primary coronary artery AHSMCs were from ATCC (USA). The supplier company guaranteed that they expressed smooth muscle α-actin and were negative for human immune deficiency virus, hepatitis B virus, hepatitis C virus, mycoplasma, bacteria, yeasts, and fungi. For first expansion purposes, the Vascular Cells Basal Medium (ATCC^®^ Cat. no. PCS-100-030) with the Vascular Smooth Muscle Growth Kit (ATCC^®^ Cat. no. PCS-100-042) was used as suggested by the seller. The Growth Kit holds recombinant human (rh) basic FGF, rh insulin, rh EGF, ascorbic acid, L-glutamine, and foetal bovine serum (FBS). For the experiments, AHSMCs’ culture medium was DMEM (89% *v*/*v*; Life Technologies Italia, Monza, Italy) supplemented with heat-inactivated (at 56 °C for 30 min) exosome-depleted FBS (10% *v*/*v*; Life Technologies Italia), and penicillin–streptomycin solution (1% *v*/*v*; Lonza, Rome, Italy). 

Microvascular HECs isolated from adult skin capillaries were from Cell Applications, Inc. (Cat. no. 300-05a, San Diego, CA, USA). The seller pledged that such cells were free from bacteria, yeasts, fungi, and mycoplasma, and expressed the Factor VIII-related antigen. HECs were grown in Endothelial Cell Growth Medium (Cat. no. C-22010, PromoCell GmbH, Heidelberg, Germany). For the experiments, HECs were kept in Endothelial Cell Basal Medium (Cat. no. C-22210, PromoCell) supplemented with heat-inactivated (at 56 °C for 30 min) exosome-depleted FBS (10% *v*/*v*; Life Technologies Italia), and penicillin–streptomycin solution (1% *v*/*v*; Lonza, Italy).

To ensure that the exosomes under study came solely from the AHSMCs, FBS was first heat-inactivated (at 56 °C for 30 min) and next centrifuged twice at 100,000× *g* for 120 min at 4 °C in an Optima TLX ultracentrifuge (Beckman, Indianapolis, IN, USA) using the type TLA 100.3 minirotor before its addition to the experimental media [16]. 

### 2.4. Intravital AHSMCs Staining and In Vitro Culture

Prior to their experimental use, 3rd or 4th passage AHSMCs were counted using a Handheld Automated Cell Counter (Scepter^TM^, Merck, Darmstadt, Germany) according to the seller’s instructions. To highlight the C/HE-3D-SFnws-attached cells, just prior to seeding 2.0 × 10^6^ AHSMCs were intravitally stained with the green fluorescent lipophilic membrane dye (tracer) DiOC_18_(3) (3,3′-Dioctadecyl oxacarbocyanine perchlorate (Thermo Fisher Scientific, Milan, Italy) with maximal fluorescence excitation at 484 nm and emission at 590 nm wavelengths. DiOC_18_(3) was dissolved in DMSO and used to intravitally stain AHSMCs according to the seller’s instructions. For each experiment, four equal aliquots (5 × 10^5^ each) of pre-stained AHSMCs were carefully seeded onto as many C/HE-3D-SFnws scaffolds placed inside separate 4-well, multi-dish polystyrene culture plates (Cat. No. 176597, Nalge Nunc International). For comparative purposes, equal aliquots (5 × 10^5^ cells each) of prestained AHSMCs were seeded in parallel onto four identical polystyrene Petri dishes. The cell cultures of both groups were incubated for 15 days at 37 °C in a 95% *v*/*v* air, 5% *v*/*v* CO_2_ atmosphere. AHSMCs were regularly seen under an inverted fluorescence microscope (IM35, Zeiss, Oberkochen, Germany) equipped with proper excitation and emission filters. All the next procedural steps were the same for the control group and the experimental group. Pictures were taken using a CP12 digital camera (Optika Microscopes, Società a responsabilità limitata, Ponteranica, BG, Italy).

### 2.5. AHSMCs’ Total DNA Assay

To estimate the cell growth on C/HE-3D-SFnws scaffolds, DNA cellular contents were calculated by the Quant-iT PicoGreen dsDNA Kit (Thermo Fisher Scientific). Three specimens of AHSMCs cultured on C/HE-3D-SFnws were assessed at experimental days 3, and 15 (Figure 1e), respectively. After washing the cells in PBS, 8 mL deionized water was added to the wells to detach and lyse the cells. Next, repeated vortexing and twice freezing–thawing improved cells lysis. Then, the DNA amounts were fluorometrically measured at excitation 480 nm and emission 520 nm wavelengths, respectively. A standard double stranded (ds) DNA curve of known concentrations served to calibrate the fluorescence intensities in relation to cell numbers. 

### 2.6. Assay of D-glucose Consumption

Cell *D*-glucose cumulative consumption was assessed in the conditioned growth medium samples from AHSMCs cultured on 3D-SFnws by a glucose oxidase assay using the Amplex^®^ Red Glucose/Glucose Oxidase Assay Kit (Thermo Fisher Scientific) following the instructions manual. Glucose oxidase reacted with *D*-glucose to form *D*-gluconolactone and hydrogen peroxide in the presence of horseradish peroxidase. Hydrogen peroxide reacted with the Amplex Red reagent in a 1:1 stoichiometric ratio to generate the red-fluorescent product resorufin, whose intensity was recorded fluorometrically at excitation and emission wavelengths of 560 and 590 nm, respectively. 

### 2.7. Phoshoproteins Array Analysis

To assess the activation of TGF-β and NF-κB intracellular signalling pathways, the phosphorylation status of selected proteins (see Table 1) in lysates from AHSMCs grown on C/HE-3D-SFnws scaffolds or on polystyrene was analysed using the C-Series RayBio^TM^ Phosphorylation pathway profiling array (RayBiotech Inc., Peachtree Corners, GA, USA). For each experiment, AHSMCs (5 × 10^5^ cells) were carefully seeded onto as many C/HE-3D-SFnws scaffolds placed inside separate 4-well, multi-dish polystyrene culture plates (Cat. No. 176597, Nalge Nunc International) (experimental group). In parallel, equal aliquots were seeded for comparative purposes onto four polystyrene Petri dishes (diameter, 10 cm; Thermo Fisher Scientific). The cell cultures of both groups were incubated at 37 °C in a 95% *v*/*v* air, 5% *v*/*v* CO_2_ atmosphere for 15 days in DMEM (89% *v*/*v*; Life Technologies Italia, Italy) supplemented with heat-inactivated (at 56 °C for 30 min) exosome-depleted FBS (10% *v*/*v*; Life Technologies Italia), and penicillin–streptomycin solution (1% *v*/*v*; Lonza, Rome, Italy).

According to the manufacturer’s instructions, cell lysates were collected from both groups by solubilizing the cells in 1X Lysis buffer (RayBiotech Inc.) added with protease inhibitor and phosphatase inhibitor cocktails. The sample protein concentrations were assessed via Bradford’s method. Briefly, each membrane supporting a different antibody array was blocked with Intercept^®^ TBS-blocking buffer (LI-COR Biosciences GmbH, Bad Homburg vor der Hohe, Germany) for 60 min at room temperature and then incubated with 50 μg of protein lysate, overnight at 4 °C. After washing, the detection antibody cocktail was added during a 2 h incubation at room temperature, followed by 1 h incubation with IRDye^®^ 800 CW-conjugated anti rabbit antibody (1:3000 in Intercept^®^ TBS-blocking buffer (LI-COR Biosciences GmbH) plus 0.2% *v*/*v* Tween-20). The positive signals of the phosphorylated proteins were acquired with an Odissey^®^ (LI-COR Biosciences GmbH) scanner and their densitometric values quantified using the Image Studio^®^ (version 5.2) software package (LI-COR Biosciences GmbH). Finally, the results were (i) processed as integrated intensity absolute values; or (ii) normalized to the maximal integrated density obtained in each single array. In keeping with Neradil et al. [49], these two processing modes resulted in identical phosphorylation profiles for each of the examined proteins. Hence, the results were expressed as the means ± SDs of three distinct experiments.

### 2.8. Isolation, Characterization, and Quantification of Exosomes

AHSMCs-conditioned media of both experimental and control groups were collected at 72 h intervals between day 3 and 15 and centrifuged at 2000× *g* for 30 min at 4 °C to remove cells and debris. The resulting supernatants were stored at −80 °C for later analysis. After thawing, the supernatants belonging to each group were pooled together and the corresponding total exosomal fractions were extracted using the Total Exosome Isolation Reagent No. 4478359 for cell culture media (provided by Thermo Fisher-Invitrogen, USA) according to the supplier’s protocol with some modifications. Briefly, the supernatants were centrifuged at 15,000× *g* for 30 min and next mixed with the proprietary reagent, incubated overnight at 4 °C, and afterward centrifuged again at 10,000× *g* for 90 min at 4 °C. The final pellets held the exosome fractions. This procedure has been compared with others and its validity confirmed [50]. The total proteins of the exosome fractions were quantified via Bradford’s method. The marker-based assessments of the exosomal preparations were performed using ELISA kits detecting the CD9 (ExoTEST^TM^, HansaBio Med, Tallinn, Estonia) and CD81 markers (ExoELISA-ULTRA CD81, System Biosciences, Palo Alto, CA, USA). Notably, CD9 and CD81 are members of the transmembrane-4 superfamily proteins intensely expressed also by the exosomes of vascular SMCs [51]. Thereafter, equal exosomal particle numbers (i.e., 1.04 × 10^11^) quantified via ELISA kits for CD9 and CD81 markers from the experimental (C/HE-3D-SFnws) and the control (polystyrene) groups were used in parallel for further processing. 

### 2.9. AGFs Carried by AHSMC-Released Exosomes

The AGFs carried by exosomes were quantified with the Human Angiogenesis Antibody Array C1000 (RayBiotech) according to the manufacturer’s protocols. Briefly, equal amounts of exosomal proteins of the control and experimental group were diluted in 2.0 mL PBS and next incubated with the antibody arrays, which had been pre-treated for 30 min with Intercept^®^ TBS-blocking buffer (LI-COR). After an overnight incubation at 4 °C and a thorough washing, the array membranes were incubated for 2 h with 1.0 mL of a mix of array-specific biotin-conjugated primary antibodies, diluted 1:250 in Intercept^®^ TBS-blocking buffer. Finally, the membranes were incubated at room temperature for 1 h with 2.0 mL of DyLight800-conjugated streptavidin (LGC Clinical Diagnostics’ KPL, Gaithersburg, MD, USA), diluted 1:7500 in Intercept^®^ TBS-blocking buffer. The positive signals of the various AGFs were acquired with an Odissey^®^ scanner (LI-COR Biosciences GmbH) and their densitometric values quantified by using the Image Studio^®^ software package (version 5.2, LI-COR Biosciences GmbH). The intensity values of the positive signals from each array were normalized via comparisons to corresponding positive controls. The results from three independent experiments were averaged and expressed as mean values ± SDs. This technology provided several advantages: (i) it allowed performing high-content screening using about the same sample volume as traditional ELISAs require; (ii) it improved the chances of discovering key factors while maintaining an ELISA-like sensitivity; (iii) it had a wider detection range, i.e., 10,000-fold, than typical ELISA assays (which is 100-to-1000-fold); and (iv) it owed a lower inter-array coefficient of variation of spot signal intensities (i.e., ~5–10%) than typical ELISAs do (i.e., ~10–15%).

### 2.10. In Vitro HECs’ Cultures

HECs were seeded into 24-well plates at 15×10^3^ cells/well and kept in Endothelial Cell Basal medium (Cat. no. C-22210, PromoCell) plus 10% *v/v* exosome-depleted FBS. A fluorescence CellTiter-Blue^®^ cell viability assay (Promega, Madison, WI, USA) served to calculate out HECs numbers. This assay measures the conversion of resazurin into fluorescent resorufin by metabolically active cells. The fluorescence intensity produced is directly proportional to the number of viable cells [52]. Thus, at the devised time points, HECs were incubated for 1 h at 37 °C in 500 μL of culture medium added with 50 μL of CellTiter-Blue^®^ reagent. The resulting resazurin was fluorometrically recorded using FP 6200 fluorometer (Jasco, Cremella (LC), Italy), with excitation and emission wavelengths of 560 nm and 590 nm, respectively. Twenty-four hours after plating (i.e., at experimental 0 h), a first CellTiter-Blue^®^ test was conducted to obtain baseline values of cell numbers. After obtaining baseline values (0 h), half of the wells, i.e., the experimental group, were randomly selected to be added with a medium enriched with exosomes (at a final concentration, 2 μg mL^−1^) that had been collected and quantified as detailed above. The remaining wells served as controls. The same amount of AHSMCs’ exosomes were added to the experimental group again 24 h later. Then, to assess changes in cell numbers, at 72 h the CellTiter-Blue^®^ test was performed again in wells of both groups. The fluorescence values gained were transformed into corresponding HECs numbers using an ad hoc constructed standard curve.

### 2.11. In Vitro HECs Migration Test

For migration studies, HECs were pre-labelled with fluorescent CellBrite^®^ NIR 680 dye (1 μM; Biotium, Inc., Fremont, CA, USA) and ~35 × 10^3^ HECs were seeded into a silicone Culture Insert-2 Well (Ibidi GmbH, Graefelfing, Germany) inserted inside a 12-well plate. HECs were cultured in Endothelial Cell Basal medium (Cat. no. C-22210, Promocell) fortified with 10% *v*/*v* exosome-depleted FBS and kept at 37 °C and in 5% *v*/*v* CO_2_ in air for at least 24 h to permit cell adhesion and the formation of a confluent monolayer. Then, the Culture Insert-2 Well removal left two defined cell patches, separated by a 500 μm wide gap. The culture medium was at once removed to be replaced either with a fresh medium holding exosomes (5 μg mL^−1^) released from C/HE-3D-SFnws-adhering AHSMCs or with basal medium containing 10% *v*/*v* exosome-depleted FBS (control wells). The culture plate was incubated at 37 °C and at various time points the fluorescence signals due to the cells migration into the gap were measured using an Odyssey^®^ Imager (LI-COR Biosciences GmbH). The fluorescence intensities were quantified using Image Studio^®^ software (version 5.2, LI-COR Biosciences GmbH). To this aim, at time 0 h, a rectangular shape (corresponding to the area of the gap) was set to define the detection zone for fluorescent signals and then, at various time points, the fluorescence intensity into the rectangular shape was quantified in real-time as the sum of the pixel values within the shape’s boundary. The HECs migration was expressed as percentage fluorescence values with respect to the experimental 0 h. The migration assays were conducted in triplicate and the mean ± SD values served to construct curves reflecting the time-related gap reduction in the two groups.

### 2.12. In Vitro HECs Tubes and Nodes Formation Assay

The proangiogenic properties of exosomes released from AHSMCs grown on C/HE-3D-SFnws scaffolds were assessed using HECs and the PromoKine Angiogenesis Assay Kit (Cat. No. PK-CA577-K905; PromoCell) according to the manufacturer’s instructions. Briefly, HECs were grown at 37 °C in air with CO_2_ 5% *v*/*v* until reaching about 90% confluency in Endothelial Cell Basal Medium (Cat. No. C-22210; PromoCell) added with the Supplement Mix (Cat. No C-39215; PromoCell). Next, HECs were harvested using trypsin 0.025% *v*/*v* and resuspended in Endothelial Cell Basal Medium (Cat. No. C-22210; PromoCell) fortified with 2.5% *v*/*v* exosome-depleted FBS. Then, an aliquot (50 μL) of Extracellular Matrix (ECM) solution was added to each well of a 96-well sterile cell culture plate kept on ice. Thereafter the plate was incubated at 37 °C to form a gel. After that, 20 x 10^3^ HECs suspended in 100 μL culture medium were mixed with different concentrations (1, 2, 5, 10, and 20 μg mL^−1^) of exosomes from C/HE-3D-SFnws-attached AHSMCs and directly added to each well. Controls on ECM gel received no exosomes. Finally, the plates were incubated for 5 h at 37 °C in air with CO_2_ 5% *v*/*v*. Thereafter, HECs were checked and photographed at 100× magnification under a Zeiss IM35 microscope with a CP12 digital camera (Optika Microscopes). The total mean tube lengths (in μm) and nodes numbers per microscopic field at 100× magnification were quantified via morphometric methods [53]. Triplicate results were averaged and graphed as bars showing the means ± SDs.

### 2.13. Statistical Analysis

Data were expressed as mean values ± SDs. Descriptive statistical analyses were conducted using the *Analyse-it*™ software package (www.analyse-it.com, accessed on 10 January 2022). Shapiro–Wilk’s test revealed that the data groups had a normal distribution. A one-sided Student’s *t* test served to assess the level of statistical significance differences of data of AHSMCs cultured on 3D-SFnws vs. AHSMCs cultured on polystyrene. A one-way ANOVA with post hoc Tukey’s test served for multiple comparisons of the results from endothelial tubes/nodes formation assays. Statistical significance was set at *p* value < 0.05.

## 3. Results

### 3.1. Carded/Hydroentangled (C/HE)-3D-SFnws

An earlier work from our laboratory detailed the physicochemical characteristics of the C/HE-3D-SFnws used here [16]. Briefly, the native SF microfibers are first mainly longitudinally oriented via carding and next transversally twisted via hydroentanglement technology. The upshot is a 3D nonwoven whose fabrication avoids the use of any gluing chemical (e.g., FA) while abiding by the biomechanical requirements of human soft tissues [54] (Figure 1a,b). Interestingly, from an applicative standpoint, according to the criteria of Wang et al. [55] and Thurber et al. [56], the properties of C/HE-3D-SFnws are consistent with a slow, i.e., medium-to-long term, biological breakdown that could advance the progressive repair/reconstruction of an injured/lost tissue in vivo. 

### 3.2. Growth and Metabolism of AHSMCs on C/HE-3D-SFnws vs. Polystyrene

Observations under the fluorescence microscope revealed that within 3 h of careful seeding, about 80% of the intravitally stained AHSMCs adhered to the C/HE-3D-SFnws microfibers (data not shown). These cells increased in number with time and moved not only along the SF microfibers but also into the inter-fibre voids after secreting extracellular matrix (ECM) (Figure 1c,d). In keeping with this, between day 3 and 15 of staying in vitro, the double strand (ds) DNA amount of polystyrene-stuck AHSMCs rose by 7.8-fold, while that of the C/HE-3D-SFnws-attached AHSMCs increased by 10.9-fold (+39.7%, *p* < 0.05) (Figure 1e). Additionally, during the same time lag, the cumulative *D*-glucose uptake from the growth medium on the part of polystyrene-attached AHSMCs increased by 6.1-fold while that of C/HE-3D-SFnws-bound AHSMCs rose by 8.4-fold (+35.5%, *p* < 0.05) (Figure 1f). These significant increases in growth and metabolic activities of the C/HE-3D-SFnws-bound vs. polystyrene-stuck AHSMCs happened despite both experimental groups being kept in the same incubator and nourished with equal amounts of the same exosome-depleted 10% *v*/*v* FBS growth medium. In fact, the two groups differed only in the substrate to which they adhered.

### 3.3. Signalling Pathways Activated in C/HE-3D-SFnws- vs. Polystyrene-Attached AHSMCs 

The activation of intracellular signalling pathways, as revealed by the phosphorylation of specific amino acidic sites, crucially drives cells’ functions. Thus, by using membrane-based double-antibody arrays, we assessed the relative phosphorylation—hence activity—levels of various proteins belonging to the TGF-β and NF-κB (also known as nuclear factor ‘kappa-light-chain-enhancer’ of activated B-cells) pathways in AHSMCs grown on either 3D-SFnws or polystyrene [57]. Table 1 lists the abbreviated names and the corresponding specific phosphorylation sites of the investigated proteins. Typical couples of developed array membranes for either experimental group are shown in Figure S1.

**Figure 2:**
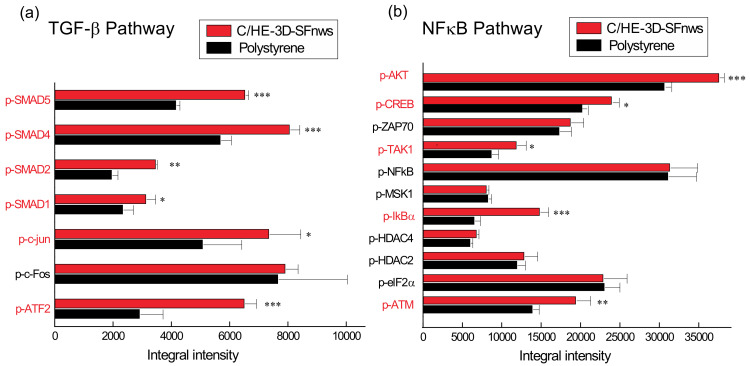
Signalling pathways activated in C/HE-3D-SFnws-attached vs. polystyrene-stuck AHSMCs. Equal amounts of total protein lysates from AHSMCs grown on either C/HE-3D-SFnws or polystyrene for 15 days in vitro were subjected to signalling pathway-specific membrane-based double-antibody arrays to assess any difference in specific sites phosphorylation of different proteins. The adhesion to C/HE-3D-SFnws significantly affects the activation of (**a**) TGF-β and (**b**) NF-κB signalling pathways in AHSMCs. Abbreviations in red highlight the increased phospho-proteins. The integrated intensity values for each couple of specific protein spots and their levels of statistical significance are shown as the mean values ± standard deviations (SDs). *, *p* < 0.03; **, *p* < 0.002; and ***, *p* < 0.0005. For technical details, consult the Section 2.7. Abbreviations: Akt/PKB, Akt/Protein kinase B; ATF-2, Activating Transcription Factor 2; ATM, Ataxia-Telangiectasia Mutated Ser/Thr kinase; c-Fos, Protoncogene c-Fos; c-Jun, Transcription factor AP1; CREB, cAMP Response Element Binding protein; eIF2α, eukaryotic translation Initiation Factor-2α; HDAC2, HDAC4, Histone DeACetylase 2/4; IκBα, NF-κB inhibitor α; MSK1, Mitogen- and Stress-activated protein Kinase 1; NF-κB, Nuclear Factor κB; SMAD1, SMAD2, SMAD4, SMAD5, Small Mother Against Decapentaplegic homolog; TAK-1, TGF-β-activated kinase 1; and ZAP70, Tyrosine protein kinase ZAP70.

Conversely, Figure 2a,b show the integrated intensity values for each couple of specific protein spots and their statistical significance. 

As the results show, the adhesion to neatly different polymeric substrates did affect the two signalling pathways investigated. In more detail:

#### 3.3.1. TGF-β Signalling Pathway

The TGF-β signalling pathway was activated more strongly in C/HE-3D-SFnws-grown than in polystyrene-stuck AHSMCs. In fact, significant increases in the phosphorylation of specific functional sites were exhibited by the downstream TGF-β signalling mediators SMAD-1 (+34%, *p* = 0.0122); SMAD-2 (+76%, *p* = 0.0026); SMAD-4 (+42%, *p* = 0.0002); SMAD-5 (+56%, *p* = 0.0001); and also by ATF-2 (+122%, *p* = 0.0005); and c-Jun (+45%, *p* = 0.03). Conversely, the specific site phosphorylation levels of c-Fos were alike (*p* > 0.05) in the two groups (Figure 2a).

#### 3.3.2. NF-κB Signalling Pathway

Five members of this pathway showed higher phosphorylation levels of specific functional sites in C/HE-3D-SFnws-grown than in polystyrene-stuck AHSMCs, i.e., TAK-1 (+36%, *p* = 0.0174); TBK-1 (+31%, *p* = 0.0092); IκBα (+126%, *p* = 0.0001); ATM (+39%, *p* = 0.002); Akt/PKB (+21%, *p* = 0.0007); and CREB (+23%, *p* = 0.0158) (Figure 2b). By contrast, the phosphorylation levels of specific sites concerning NF-κB; eIF-2α; HDAC-2; HDAC-4; and MSK-1 did not differ (*p* > 0.05) between the two groups (Figure 2b). 

Altogether, the two activated signalling pathways underlay the more intense proliferation and metabolism occurring in the C/HE-3D-SFnws-attached than in the polystyrene-stuck AHSMCs.

### 3.4. AGFs Released via Exosomes from AHSMCs Grown on C/HE-3D-SFnws vs. Polystyrene 

**Figure 3:**
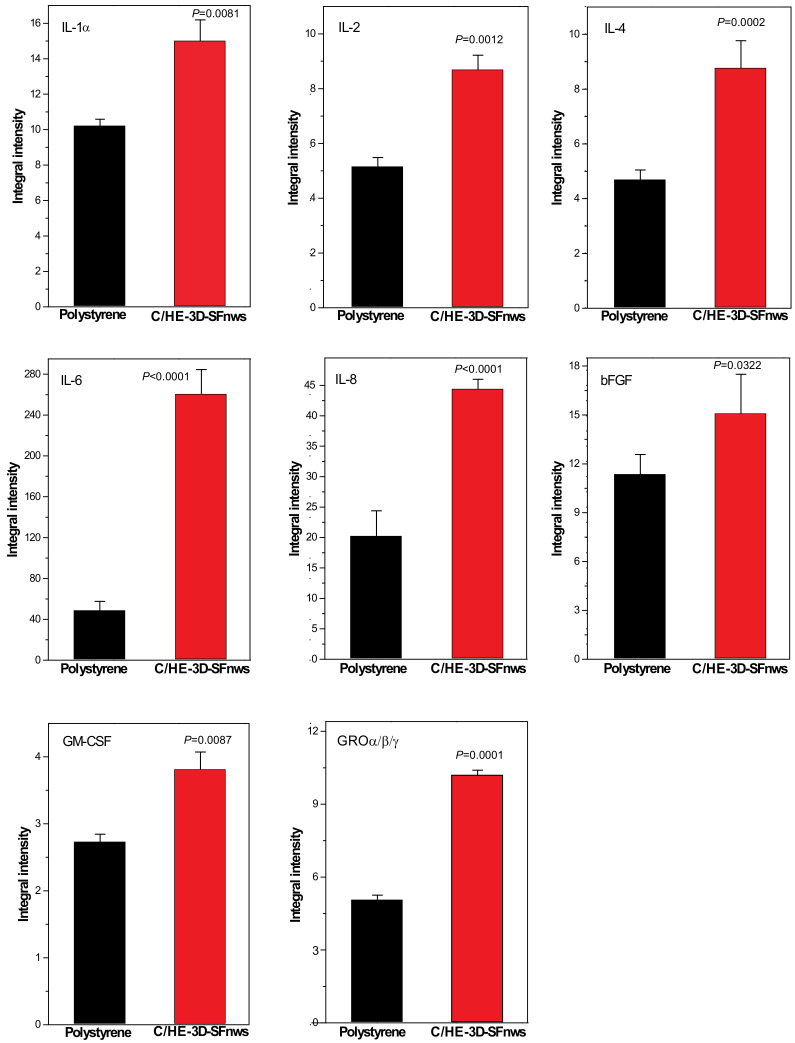
The angiogenic and trophic factors (AGFs) carried by the exosomes released from AHSMCs grown on C/HE-3D-SFnws vs. polystyrene. Equal amounts of exosomal proteins isolated from AHSMCs-conditioned pooled media samples of the two experimental groups were subjected to membrane-based double-antibody arrays, detecting the relative levels of multiple AGFs. The adhesion to C/HE-3D-SFnws significantly affected the levels of the eight exosomally carried AGFs shown in Figure. The bars are integral intensity values expressed as mean values ± standard deviations (SDs) from three distinct experiments, each conducted in duplicate. The corresponding *p* values of the differences between each couple of bars are also shown over the top of the right bars. For technical details, consult the Section 2.9.

The AGFs present in equal amounts of the pooled CD9^+^/CD81^+^ exosomes released between day 3 and 15 from AHSMCs grown on either C/HE-3D-SFnws or polystyrene were identified and quantified by means of specific membrane-based double-antibody arrays [57]. Figure S2a,b shows corresponding couples of typical array membranes of the two groups. The quantitative and statistical analysis of equivalent spots revealed that the amounts of 8 out of the 43 potentially discoverable AGFs were significantly higher (*p* < 0.05) in the exosomes released from C/HE-3D-SFnws-attached than from polystyrene-stuck AHSMCs (Figure 3). Interestingly, TGF-β was transported in alike amounts by the exosomes released from both groups (Figure S2a,b). In the C/HE-3D-SFnws group of exosomal proteins, the highest per cent increases vs. their polystyrene counterparts were those of Interleukin-6 (IL-6; +442%, *p* < 0.0001); Interleukin-8 (IL-8; +117%, *p* < 0.0001); and Growth-Regulated Oncogene (GRO)-α/-β/-γ chemokines (+100%, *p* < 0.0001). Lesser but still significant increases were observed for Interleukin-4 (IL-4; +87%, *p* < 0.0002); Interleukin-2 (IL-2; +71%, *p* <0.001); Interleukin-1α (IL-1α; +48%, *p* < 0.001); Granulocyte-Macrophage Colony-Stimulating Factor (GM-CSF; +41%, *p* < 0.0087); and basic Fibroblast Growth Factor (bFGF; +33.3%, *p* <0.033) (Figure 3). Conversely, the exosomal amounts of eight more known angiogenic compounds—Plasminogen/Angiostatin; ANGPT-2 (Angiopoietin-2); Tie-2 (Angiopoietin-1 receptor); MCP-1 (Monocyte Chemoattractant Protein-1); VEGF-D (Vascular Endothelial Growth Factor-D); VEGF-R3 (VEGF receptor 3); and TIMP-1 and TIMP-2 (Tissue Inhibitor of Metalloproteinase-1/-2)—did not significantly (*p* > 0.05) differ between the C/HE-3D-SFnws and polystyrene groups. Finally, another 27 agents were identified in similarly low amounts in the exosomes from both groups (Figure S2a,b). 

These findings are consistent with the reported angiogenic and growth-promoting effects of the more intensely expressed AGFs carried by the exosomes released from C/HE-3D-SFnws-attached AHSMCs (see Table 2 and for more details the Supplementary Materials).

### 3.5. Exosomes from C/HE-3D-SFnws-Stuck AHSMCs Powerfully Stimulate HECs to Grow, Migrate, and Make Endothelial Tubes/Nodes

Next, we assessed whether the AGFs carried by the exosomes released from the C/HE-3D-SFnws-bound AHSMCs would have any real angiogenic power by inducing cultured HECs to proliferate, migrate, and/or de novo assemble into endothelial tubes and establish nodes in vitro. 

**Figure 4:**
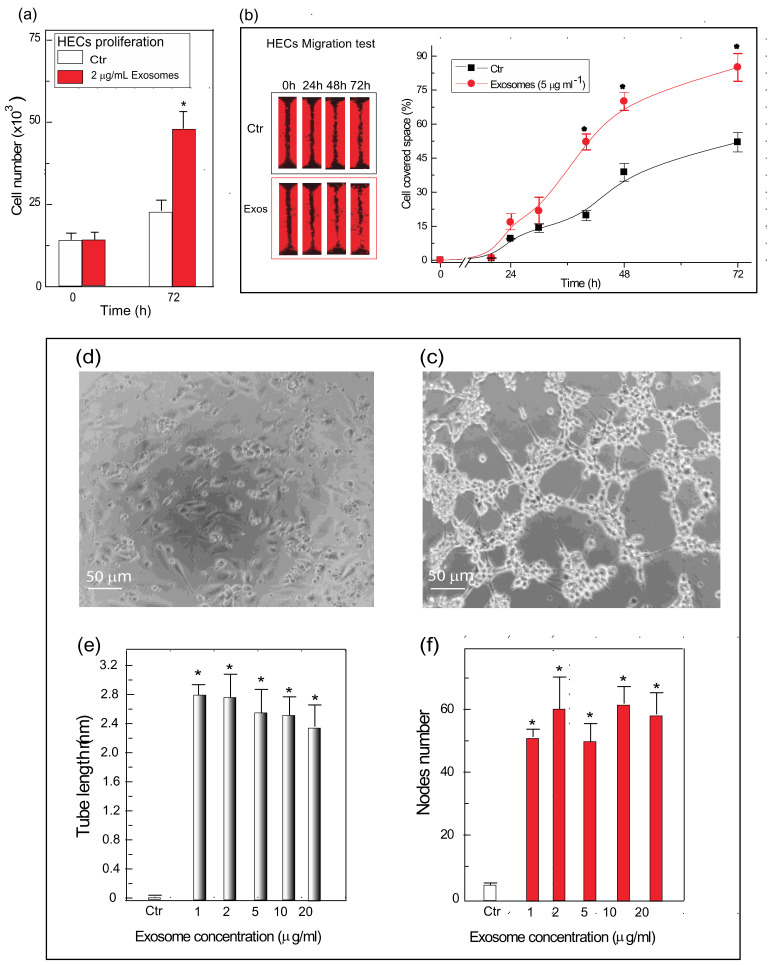
Exosomes released from AHSMCs grown on C/HE-3D-SFnws induce HECs’ proliferation, migration, and tubes and nodes formation in vitro. (**a**) Stimulation of HECs’ growth: exosomes (2 μg mL^−1^) released from C/HD-3D-SFnws-adhering AHSMCs when added to HECs cultured in Endothelial Cell Basal medium plus 10% *v*/*v* exosome-depleted FBS strongly increased the numbers of viable cells 72 h later, as revealed by CellTiter-Blue^®^ assay vs. the untreated cells (Ctr). *, *p* < 0.05 vs. Ctr. See Materials and Methods for technical details. (**b**) Stimulation of HECs’ migration: adding exosomes (5 μg mL^−1^) released from the C/HE-3D-SFnws-bound AHSMCs to HECs cultured in Endothelial Cell Growth Basal Medium fortified with 2.5% *v*/*v* exosome-depleted FBS significantly advanced their migration into the “wound area” between 24 h and 72 h as compared to untreated HECs (Ctr), as the gap’s space covered by the exosome-treated HECs was, by 72 h, about double (*p* < 0.05) that occupied by the untreated cells (Ctr). For technical details consult the Section 2.11. (**c**,**d**) Tube and nodes formation: micrograph (**c**) showing plain attached HECs 5 h after seeding into a 96-well plate onto Extracellular Matrix (ECM) gel. In each of these wells 20 × 10^3^ HECs were incubated at 37 °C in Endothelial Cell Growth Basal Medium fortified with 2.5% *v*/*v* exosome depleted FBS. No AHSMC-released exosomes were added. Micrograph (**d**) shows HECs 5 h after seeding onto ECM gel while being simultaneously exposed to increasing concentrations of exosomes released from C/HD-3D-SFnws-adhering AHSMCs, starting from 1 μg mL^−1^ to 20 μg mL^−1^; all the other conditions as in (**c**). Endothelial tube formation was strongly induced by the AHSMCs exosomes. (**c**,**d**), Phase contrast microscopy. Original magnification, 100×. (**e**,**f**) Bar graph showing the total length (in mm) of newly formed endothelial tubes (e) and the number of nodes (**f**) per microscopic field under the conditions of the test, i.e., (i) control HECs cultured on ECM gel with no addition of exosomes set free from C/HE-3D-SFnws-attached AHSMCs; and (ii) HECs cultured on ECM gel exposed to increasing concentrations of exosomes released from C/HE-3D-SFnws-attached AHSMCs. Tube formation assay was performed as detailed in the Section 2.12. The number of nodes and the total tube length (in mm)/microscopic field of 332,667 μm^2^ area were found via morphometric methods [53] on pictures taken at 100× magnification of five microscopic fields for each exosomal concentration. Triplicate results were averaged, and the bars show the means ± SDs. As compared to control HECs on Extracellular Matrix gel (Ctr), in the total absence of exosomes, the HECs treated with exosomes released from C/HE-3D-SFnws-attached AHSMCs showed, by 5 h, huge increases in endothelial tube lengths and in numbers of nodes that were dose-independent in the range evaluated. Pairwise one-tailed Student’s *t* test and one-way analysis of variance (ANOVA) with post hoc Tukey’s test were used for statistical analysis. *, *p* < 0.001 vs. Ctr. Conversely, no statistical difference (*p* > 0.05) in tube length and number of nodes at 5 h occurred within the several doses of exosomes evaluated.

First, adding exosomes (2 μg mL^−1^) to HECs cultured in exosome-depleted 10% *v*/*v* FBS medium significantly increased the numbers of viable, i.e., metabolically active cells 72 h later, as revealed by the CellTiter-Blue^®^ assay vs. their untreated counterparts (Figure 4a).

Second, adding exosomes (5 μg mL^−1^) to HECs cultured as above significantly advanced their migration into the “wound’s area” between 24 h and 72 h as compared to untreated HECs. By 72 h, the gap’s space covered by the exosome-treated HECs was about double (*p* < 0.05) that overlain by the untreated (control) cells (Figure 4b). Moreover, the mobilization of exosome-treated HECs toward other directions was also discernible in the experimental model used (not shown).

Thirdly, control (no exosomes added) HECs plated on ECM gel in 2.5% *v*/*v* exosome-depleted FBS medium formed very few endothelial tubes/nodes. Conversely, after 5 h exposure to different doses (1, 2, 5, 10, and 20 μg mL^−1^) of exosomes, the ECM gel-plated HECs formed quite extensive tubular networks, interconnected by a considerable number of nodes. The increases in lengths of the endothelial tubes (by 35-fold to 42-fold vs. untreated controls, *p* < 0.001) and in numbers of nodes (by 11-fold to 13-fold vs. untreated controls, *p* < 0.001) per microscopic field were quite conspicuous and alike (*p* > 0.05) for each of the doses assessed (Figure 4c–f).

Therefore, the AGFs carried by the exosomes released from 3D-SFnws-attached AHSMCs exerted effective mitogenic, mobilizing, and angiogenic activities when added to HECs cultured in vitro.

## 4. Discussion

The C/HE-3D-SFnws scaffolds we presently used consist of native SF microfibers in β-sheet form isolated from domesticated *Bombyx mori* silkworms. These C/HE-3D-SFnws [16] mark a technological evolution with respect to the earlier FA-crosslinked [10] and C/N-3D-SFnws [11]. Indeed, both these earlier prototypes performed quite well in terms of biocompatibility, host response, and the engineering/regeneration of a reticular connective tissue (see also below) [10,11]. However, from a biomechanical standpoint, both earlier kinds of SFnws were unsatisfactory, which blunted their potential clinical application. While the FA-crosslinked SFnws remained somehow stiff, even after a long-lasting hydration, the C/N-3D-SFnws were thin (i.e., 130 μm thick) as their production required short (<25 mm) SF fibres as the starting material and hence they could be misshaped easily when handled [11,16]. Conversely, the carding/hydroentanglement processing of the present scaffolds required longer SF fibres (> 50 mm) thereby producing thicker and mechanically more robust structures. Therefore, citing Hu et al. [16] the novel C/HE-3D-SFnws “*maintain all the most appreciable characteristics of the carded-needled prototype* (i.e., *softness, lightness, interconnected porosity); display an outstanding handling stability (can be safely cut to realize any required size and shape); and at the same time provide the opportunity for modulating the biomechanical responses over a wider range of stress and strain values*”. Moreover, Hu et al. [16] also stated that being anisotropic, the C/HE-3D-SFnws scaffolds “*are particularly suited to fulfil* human *soft tissues mechanical needs engendered by directionally projected lines of force*”. Therefore, the C/HE-3D-SFnws characteristics appoint them as useful candidates for the guided engineering/regeneration and repair of injuries suffered by human soft tissues, hollow viscera walls and vessels included.

To be successful, an implanted biomaterial scaffold crucially requires an efficient neovascularization. When it misses this target, it is inexorably bound to fail. In earlier works, we showed that once grafted into the subcutaneous tissue of C57/BL6 mice, both the FA- and C/N-3D-SFnws underwent a fast neovascularization [10,11]. A more recent study using the same C/HE-3D-SFnws as used here showed that the scaffold-attached HDFs released exosomes loaded with surpluses of a dozen distinct AGFs, which powerfully stimulated HECs to abundantly produce endothelial tubes in vitro [16]. 

Fibroblasts are important, but not the sole constituents of the dermal and subcutaneous layers of the skin and of other connective tissue types. An added cell kind, the SMCs, populates the contractile layers or *tunicae* of hollow viscera, vessels included. Under normal conditions, adult SMCs express sets of specific proteins by which they regulate visceral and vascular contractile tone; secrete ECM components; and keep a quite low proliferation rate. However, SMCs never undergo terminal differentiation, as they own a distinct phenotypic plasticity [82]. In the adult vessel walls, SMCs of the epithelioid/synthetic-secretory and potentially migratory/proliferative phenotype prevail in the *tunica intima,* while SMCs of the spindle-shaped contractile phenotype hold sway in the *tunica media*; however, a certain number of SMCs of the less abundant phenotype is always present in either *tunica* [83]. In vitro and in physiological and pathological or harmful conditions in vivo, SMCs shift from their quiescent/spindle-shaped/contractile phenotype to their epithelioid/synthetic-secretory/migratory/proliferative phenotype. At variance with the former, the latter phenotype drives a vascular and/or visceral wall remodelling and is more prone to apoptosis [82,84,85]. In vivo, such a phenotype shift can result in neointima formation, stent occlusion, atherosclerosis, thrombosis, and asthma [86,87,88,89]. 

Wild-type or genetically modified rodent SMCs have often served as (mostly vascular) disease models [90,91]. However, animal SMCs, although undeniably useful, do not perfectly model human SMCs due to species-specific discrepancies in macroscopic anatomy, chromosome complement, genomic function, biochemistry, metabolism, and mechanical factors [30,91]. These limitations fully justify experimental studies into human SMCs as the most relevant models of human pathobiology. However, as human SMCs have different embryological origins, one should be wary of extrapolating the findings gained from SMCs of one origin to all the other SMCs [29,30]. Although the coronary artery AHSMCs used in this study have the same embryological origin as other visceral SMCs, the translatability of the present results to the latter cells requires further assessments.

The results of our earlier and present in vitro studies have repeatedly confirmed the high biocompatibility of 3D SFnws in relation, not only to HDFs and HECs, but also to AHSMCs. As well consistent with our earlier observations, SF was superior to polystyrene as an AHSMCs’ adhesion substrate [10,11,15,16,38,92]. Various reports have proven that biomaterial substrates owning patterned or microtopographic structures advanced HECs’ adhesion and proliferation more than nonpatterned ones did [93,94]. We recall here that 3D-SFnws have a patterned structure made of microfiber wisps separated by voids or grooves. Such patterning favoured the first adhesion of the AHSMCs, HDFs, and HECs. Subsequently, the same 3D-SFnws microfibers functioned as guides along which the proliferating AHSMCs first migrated and later invaded and colonized the intervening voids [10,11,15,16,92]. Our results revealed that higher increases in dsDNA (i.e., cell numbers) and glucose consumption occurred between day 3 and 15 in the AHSMCs attached to C/HE-3D-SFnws than to polystyrene. The same AHSMCs also showed higher growth and metabolic rates when stuck on a 3D SF nanofiber-microfiber-based vessel model [38]. In sharp contrast, unidirectionally aligned topologically patterned 2D SF films were reported to decrease SMCs’ mitotic rate and to promote their transition from a synthetic/highly proliferative to a contractile/lowly proliferative phenotype [95,96]. At the root of these discordant findings might be differences in SF purity, age of SMCs donors, SMCs embryological origins, and passage numbers in vitro. Moreover, we recall here that when FBS, which carries various survival and growth factors, is added to the culture medium, the mechanism of contact inhibition of growth no longer regulates AHSMCs’ proliferation [88]. At any rate, we used the very same growth medium fortified with identical per cent fractions of the same batch of exosome-depleted FBS to cultivate AHSMCs on C/HE-3D-SFnws or on polystyrene. Therefore, the exosome-depleted FBS we added did not affect the observed differences proliferation rates between AHSMCs grown on SFnws or polystyrene. Instead, the environmental context, i.e., the direct AHSMCs contact with SF or polystyrene, and/or the unlike autocrine effects brought about by the different AGFs amounts carried by AHSMC-released exosomes significantly affected the cell growth and metabolism of either experimental group. 

Consistent with such a view, we found that AHSMCs produced and released discrete amounts of TGF-β, part of which was carried by exosomes (Figure S2, panel [a]). This TGF-β may have been one of the agents activating TAK-1 (also known as TGF-β-activated kinase 1) an evolutionarily conserved MAPK kinase kinase (MAPKKK) family member placed upstream the TGF-β intracellular signalling pathway [97,98]. However, as TGF-β levels in the exosomes and the growth medium (data not shown) of the two experimental groups were alike (*p* > 0.05), other agents must have played a part in TAK-1 activation. Besides TGF-β, TAK-1 is also activated by TNF, IL-1, Toll-like receptors (TLRs) ligands, and various kinases (see [99] for further details). The observed increase in the Ser^412^ phosphorylation is indeed pivotal for TAK-1 activation [100]. It might have been related via an autocrine mechanism to the increased IL-1α content in the exosomes from SFnws-cultured AHSMCs (see also below). Moreover, both the catalytic subunit α of PKA (also known as cyclic AMP-dependent protein kinase A), or the cyclic AMP-controlled PRKX (or serine/threonine X-linked protein kinase) phosphorylate TAK-1 at Ser^412^ activating it [100]. That an increased activity of cyclic AMP-related kinases was going on in the SF-stuck AHSMCs is consistent with the heightened Ser^133^ phosphorylation and activation of the CREB (also known as cyclic AMP response element-binding) protein (Figure S1, panel (b)), which could be related to the increased proliferative activity of these cells [101]. Going back to the TGF-β pathway, the higher phosphorylation/activation of SMAD-1 and SMAD-5, two of the downstream TGF-β signalling mediators, suggests the involvement of both in the intensified growth and metabolism proper of the SF-attached AHSMCs [102]. The heightened phosphorylation/activation of SMAD-4 too—the cofactor forming complexes with any other SMAD homo- and heterodimers to transfer them into the nucleus and accordingly change gene expression [103]—supports an ongoing, more intense activity of the TGF-β signalling pathway in the SF-stuck AHSMCs. On the other hand, the less intense increase in phosphorylation/activation of the SMAD-2 mediator suggests that a fraction of the AHSMCs might have started shifting from the proliferative/secretory to the quiescent/contractile phenotype. This view agrees with the report that vascular SMCs cultured as multi-layered aggregates eventually entered a resting phenotype [104]. Moreover, the increased phosphorylation/activation of ATF-2, a protein encoded by a SMAD-targeted gene [105] belonging to the AP-1 family of transcription factors, which may also bind SMADs, further confirms that an intensification of various TGF-β pathway-related activities was occurring in the SFnws-adhering AHSMCs. AP-1 transcription factors and SMAD proteins do reciprocally interact thus modulating their respective effects in complex ways [106]. 

The said phosphorylation/activation of TAK-1 may also reveal a crosstalk between the TGF-β and NF-κB signalling pathways in the SFnws-adhering AHSMCs. In fact, TAK-1 signalling is all-important for the NF-κB’s canonical activation mediated by signals from various receptors including IL-1 receptor I (IL-1RI∙IL-1α; see also above) [107,108,109], and by stimuli linked to adaptive immunity [110,111,112,113]. Working TAK-1 phosphorylates at Ser^177^ and Ser^181^ and activates the IKKβ kinase. In turn, the activated IKKβ phosphorylates at Ser^32^ (and Ser^36^) IκBα. When not phosphorylated at such sites, IκBα binds and masks the nuclear localization signals (NLS) of the NF-κB factors Thus, the inactive IκBα∙NF-κB transcription factor complexes stay sequestered in the cytoplasm [114]. Additionally, inside the nucleus unphosphorylated IκBα hinders the binding of the dimeric NF-κB transcription factors to their specific target gene sequences [115]. However, the phosphorylation of IκBα at Ser^32^ (and Ser^36^) by IKKβ targets IκBα to the S26 proteasome for degradation while letting the cytoplasmic NF-κB transcription factors to enter the nucleus regulating cell proliferation, adhesion, mobility, and reactive oxygen species (ROS) elimination [116,117] in the C/HE-3D-SFnws-stuck AHSMCs. In summary, our results suggest the existence of a circular positive interaction by which exosomally released cytokines such as IL-1α and IL-8 supported the more vigorous growth- and migration-related activities sustained by TAK-1/NF-ĸB/CREB signalling that in turn increased the expression of IL-1α and IL-8.

Interestingly, the present results also revealed a more intense phosphorylation/activation of the ubiquitously expressed ATM (also known as Ataxia-Telangiectasia Mutated), a serine/threonine kinase member of the PIKK (phosphatidylinositol-3 kinase-related kinases) protein family [118]. In the absence of any DNA damage [119,120] an increased ATM activity importantly participates in multiple cellular events (detailed in [121,122,123,124,125]). Moreover, ATM also phosphorylates and activates Akt/PKB at Ser^473^, an intensified event that occurred in the SF-stuck AHSMCs, which may have positively affected their glucose uptake, protein synthesis, survival, and proliferation/differentiation [126,127,128].

Within the walls of vessels ECs and SMCs are close neighbours and reciprocally interact under physiological and pathological circumstances [129,130,131]. To build functioning blood vessels, tube forming ECs secrete TGF-β and PDGF to recruit SMCs [132]. Although Tang et al. [133] reported discrepant data, our results clearly show that 3D-SFnws-stuck AHSMCs did advance vasculogenesis by releasing exosomes that conveyed significant amounts of multiple angiogenic/growth factors. These findings consist with reports showing that the exosomes released from 3D cell culture models improved angiogenesis more effectively than those from 2D cultures [134]. However, we wish to stress that it is the *combination* of all the exosome- transported AGFs in their proper ratios that will optimally advance the three processes proper of neovascularization, i.e., vasculogenesis, angiogenesis, and arteriogenesis. 

AHSMCs can be induced by a variety of stimuli to synthesize several cytokines and chemokines [135]. Therefore, a further potential benefit of our earlier and present results is that, after proper purification and standardization, the exosomes produced by 3D-SFnws bioreactors hosting AHSMCs or fibroblasts could find beneficial therapeutic applications as vascularization and cell growth stimulants, thus advancing injury healing and repair in clinical settings [16,26].

## 5. Conclusions and Future Perspectives

The present results reveal that AHSMCs cultured on the same C/HE-3D-SFnws as above released exosomes carrying 15 AGFs, of which 8 were significantly enriched. The AHSMCs exosomes also powerfully stimulated HECs to grow, migrate, and form dense tubes and nodes in vitro. Interestingly, only six of these AGFs, i.e., GRO-α/CXCL1, GRO-β/CXCL2, GRO-γ/CXCL3, IL-8/CXCL8, IL-4, and IL-1α, were significantly enriched in the exosomes released from both AHSMCs and HDFs [16] cultured on C/HE-3D-SFnws. However, it is worth noting that the exosomes released from the two cell types also transported discrete amounts of 10 other AGFs, e.g., MCP-1 (Monocyte chemoattractant protein-1), VEGF-D, ANGPT-1/-2, Tie2 (ANGPT-1 receptor), Angiostatin, uPAR (CD87), MMP-1/-9 (Matrix Metalloproteinase-1/-9), and TIMP-1/-2. 

Finally, another score of compounds—i.e., TGF-β1, IGF-1, PDGF-BB, GM-CSF, I-309 (CCL-1), IL-10, IL-1β, Endostatin, I-TAC (Interferon-inducible T cell Alpha Chemoattractant), Leptin, PLGF (Placental Growth Factor), RANTES (Regulated upon Activation, Normal T Cell Expressed and Presumably Secreted chemokine), TNF-α, TPO (Thrombopoietin), VEGF-A, MCP-2, MCP-4, and PECAM-1 (Platelet Endothelial Cell Adhesion Molecule-1)—were conveyed solely by the exosomes released from the C/HE-3D-SFnws-adhering AHSMCs (Figure S2) being undetectable in those released from their HDFs counterparts [16]. 

Altogether, these observations show that patterns of enriched or not enriched AGFs released via exosomes by SF-stuck AHSMCs and HDFs exhibit not only substrate-type- but also cell type-specificity. The latter could affect the characteristics of the neovascularization AHSMCs and HDFs, respectively induced—a topic well worth investigating further. In this regard, D’Amore and Smith [136] showed that, according to their origin in small or large vessels, HECs’ responses to a set of growth factors exhibited quantitative and qualitative differences. 

Altogether, our results strengthen the view that once grafted in vivo 3D-SFnws can decidedly advance their own vascularization by inducing the colonizing human fibroblasts and SMCs to release loads of enriched AGFs via exosomes. We posit that an alike mechanism operated when we grafted FA- and C/N-3D SFnws into the subcutaneous tissues of mice in vivo [10,11]. 

Altogether, our results stress the importance of the AGFs in protein form, transported by the exosomes released from 3D-SFnws-attached AHSMCs and from HDFs [16]. The role(s) of any angiogenic RNAs carried by exosomes from the same sources will be addressed by future studies. The crucial insights into the interactions between SFnws and nontumorigenic AHSMCs, HDFs, and HECs we brought to light bode well for prospective applications of SF-based properly structured scaffolds in human (and even veterinary) clinical settings. 

## Figures and Tables

**Table 1:** Phosphorylated proteins investigated via membrane-based double antibody arrays.

Abbreviations	Names in Extenso	Phosphorylation Site (s)	References *
**Akt/PKB**	Akt/Protein Kinase B	Ser473	102–104
**ATF-2**	Activating Transcription Factor-2	Thr69/Thr71	81
**ATM**	Ataxia-Telangiectasia Mutated Ser/Thr kinase	Ser1981	82
**c-Fos**	Protein of transcription factor AP1	Thr232	
**c-Jun**	Protein of transcription factor AP1	Ser73	82
**CREB**	cyclic AMP Response Element Binding protein	Ser133	77
**eIF-2α**	eukaryotic translation Initiation Factor-2α	Ser51	
**HDAC-2**	Histone Deacetylase-2	Ser394	
**HDAC-4**	Histone Deacetylase-4	Ser632	
**IκBα**	NF-κB Inhibitor α	Ser32	90
**MSK-1**	Mitogen- and Stress-activated protein Kinase	Ser376	
**NF-κB**	Nuclear Factor-κB	Ser536	85
**SMAD-1**	Small Mother Against Decapentaplegic homolog-1	Ser463/Ser465	78–80
**SMAD-2**	Small Mother Against Decapentaplegic homolog-2	Ser245/Ser250/Ser255	
**SMAD-4**	Small Mother Against Decapentaplegic homolog-4	Thr277	
**SMAD-5**	Small Mother Against Decapentaplegic homolog-5	Ser463/Ser465	
**TAK-1**	TGF-β-Activated Kinase 1	Ser412	76
**ZAP-70**	ZAP-70 Tyrosine protein kinase	Tyr292	

* These references relate only to proteins whose phosphorylation levels were significantly changed.

**Table 2:** The main trophic and angiogenic features of each of the enriched AGFs conveyed by the exosomes released from 3D-SFnws-adhering AHSMCs.

AGF	Trophic and Angiogenic Actions
**IL-1α**	promotes angiogenesis in vivo by inducing VEGF’s synthesis [58]; activates the VEGF∙VEGFR-2 signalling pathway; stimulates Platelet-Derived Growth Factor (PDGF)’s A chain [59], and bFGF expression [60]; induces its own expression in vascular SMCs, exerting autocrine growth-stimulatory effects [61].
**IL-2**	affects the permeability of ECs [62] and promotes ECs angiogenesis through the α and β IL-2 receptors; stimulates angiogenesis in animals and tube formation in HUVECs [63]; enhances SMCs responsiveness to angiotensin II [64]; in cooperation with IL-1α potentiates human SMCs proliferation [65].
**IL-4**	increases the expression of vascular cell adhesion molecule (VCAM)-1, IL-6, and MCP-1 [66]; induces cytoskeletal rearrangements both in HUVECs and in human coronary artery ECs [67]; acts as a mild mitogen for both macro- and microvascular ECs [68,69,70].
**IL-6**	exerts autocrine growth-stimulating effects on vascular SMCs inducing endogenous PDGF’s production [71]; support ECs’ cell proliferation and mobility [72].
**IL-8** **GRO-α/β/γ**	by sharing the evolutionary ‘ELR’ motif they all powerfully promote angiogenesis even in the absence of inflammation [73,74].
**bFGF**	regulates both angiogenesis and arteriogenesis (reviewed in [75]); enhances ECs and SMCs proliferation [76]; regulates vascular remodelling and the proliferation of human dermal microvascular ECs [77,78].
**GM-CSF**	supports ECs leading to the formation of endothelial capillaries [79]; stimulates the migratory repair of mechanically wounded ECs monolayers [80]; stimulates angiogenesis, neovascularization, and arteriogenesis [81].

## Data Availability

The datasets of this study are available upon request to the corresponding authors.

## Supplementary Materials

The following supporting information can be downloaded at: https://www.mdpi.com/article/10.3390/polym14040697/s1, Figure S1: Developed double-antibody array membranes and array maps of human phosphorylation signalling pathways. Figure S2: Developed double-antibody array membranes. Details on the trophic and angiogenic actions of the AGFs conveyed in increased amounts by the exosomes released from C/HE-3D-SFnws-stuck AHSMCs.

## Abbreviations

3D-SFnws	three-dimensional silk fibroin nonwovens
AGFs	angiogenic and growth factors
AHSMCs	adult human smooth muscle cells
Akt	protein kinase B or PKB
ANGPT-1/2	angiopoietin-1/2
ANOVA	analysis of variance
ATM	ataxia-telangiectasia mutated Ser/Thr kinase
bFGF	basic Fibroblast Growth factor
c-Fos	component of AP1 transcription factors
c-Jun	component of AP1 transcription factors
CREB	cyclic AMP response element binding protein
C/HE	carding/hydroentanglement
C/N	carding/needling
DiOC18(3)	(3,3′-Dioctadecyl oxacarbocyanine perchlorate or DiO
(ds)DNA	double strand DNA
ECM	extracellular matrix
ECs	endothelial cells
eIF-2α	eukaryotic translation initiation factor-2α
ELISA	enzyme-linked immunosorbent assay
FA	formic acid
FBS	foetal bovine serum
GM-CFS	granulocyte macrophage-colony stimulating factor
GRO-α/-β/-γ	growth-regulated oncogene-α/-β/-γ (or CXCL1/2/3)
HADAC	histone deacetylase
HDFs	human dermal fibroblasts
HECs	human ECs
IKK	IkappaB kinase
IκBα	nuclear factor of kappa light polypeptide gene enhancer in B-cells inhibitor, alpha
IL-	interleukin-
MAPK	mitogen-activated protein kinase
MCP-1	monocyte chemoattractant protein-1 (or CCL2)
MMP	matrix metalloprotease
MSK-1	mitogen, and stress-activated protein kinase
NF-κB	nuclear factor κB
rh	recombinant human
SD	standard deviation
SF	silk fibroin
SMAD	small mother against decapentaplegic homolog
SMCs	smooth muscle cells
TAK-1	TGF-β-activated kinase-1
TCF/LEF	T-cell factor/Lymphoid enhancer factor
TGF-β	transforming growth factor-beta
Tie-2	ANGPT-1 receptor
TIMP-1/2	tissue inhibitor of metalloproteinases-1/2
TLR	Toll-like receptor
uPA	urokinase-like plasminogen activator
uPAR	uPA surface receptor
VEGF	vascular endothelial growth factor
VEGF-R	VEGF receptor
