# Supplementary material for: Adult Human Vascular Smooth Muscle Cells on 3D Silk Fibroin Nonwovens Release Exosomes Enriched in Angiogenic and Growth-Promoting Factors
Source: Polymers (Basel). 2022 Feb 11;14(4):697. doi: 10.3390/polym14040697 (PMC8875541; doi:10.3390/polym14040697)
Supplement: Supplementary file 1 [file polymers-14-00697-s001.zip › polymers-1582093-supplementary.pdf]

polymers

MDPI

Supplementary Materials

Adult human vascular smooth muscle cells on 3D silk fibroin nonwovens release exosomes enriched in angiogenic and growth-promoting factors

Peng Hu[1,2 †], Anna Chiarini[1*†], Jun Wu[3], Zairong Wei[2], Ubaldo Armato[1,3], Ilaria Dal Prà[1,3,*†]

[1]Human Histology & Embryology Section, Department of Surgery, Dentistry, Pediatrics & Gynecology, University of Verona Medical School, Verona, Italy; [2]Department of Burns & Plastic Surgery, The Affiliated Hospital of ZunYi Medical University, ZunYi City, , People's Republic of China; Department of Burns and Plastic Surgery, Second People's Hospital, University of Shenzhen, Shenzhen, , People's Republic of China

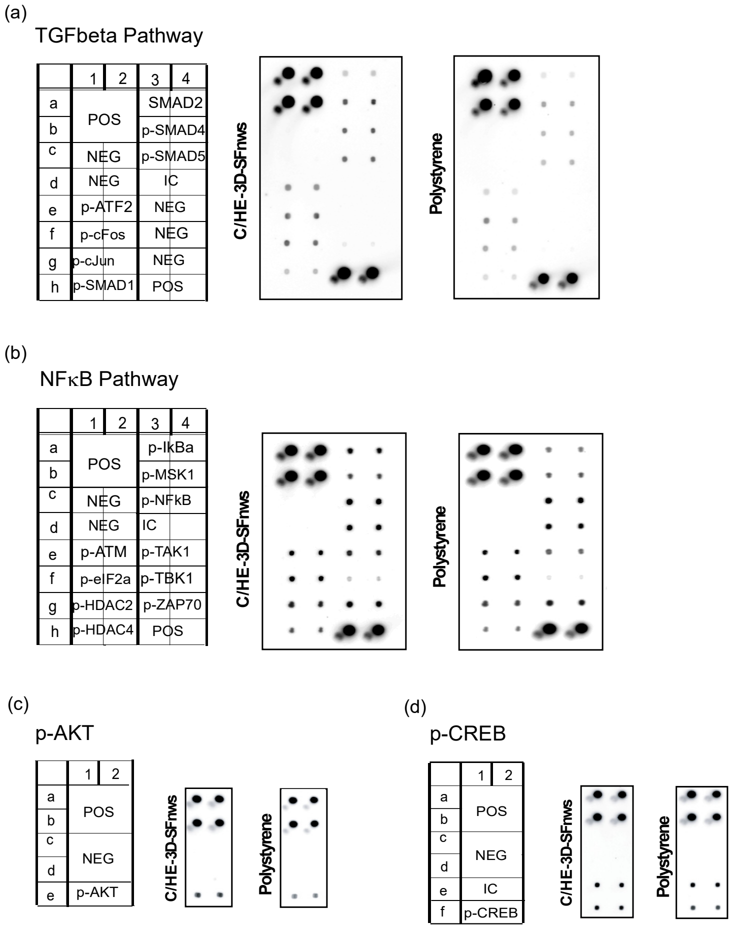

Figure S1. Typical developed double-antibody array membranes and array maps of human phosphorylation signalling pathways. (**a**) TGF-β. (**b**) NFκB signalling pathways. (**c**) Phospho (p)-AKT and (**d**) phospho (p)-CREB spots. Equal amounts of total protein lysates from AHSMCs grown on either C/HE-3D-SFnws or polystyrene for 15 days *in vitro* were subjected to signalling pathway-specific membrane-based double-antibody arrays assessing the differences in specific sites phosphorylation of proteins belonging to distinct signalling pathways. For technical details consult the *Materials & Methods. Abbreviations*, Akt/PKB, Akt/Protein kinase B; ATF-2, Activating Transcription Factor 2; ATM, Ataxia-Telangectasia Mutated Ser/Thr kinase; c-Fos, Protooncogene c-Fos; c-Jun, Transcription factor AP1; CREB, cAMP Response Element Binding protein; eIF-2α, eukaryotic translation Initiation Factor-2a; HDAC-2, HDAC-4, Histone DeACetylase -2/-4; IκBα, NF-κB inhibitor α; MSK1, Mitogen- and Stress-activated protein Kinase 1; NF-κB, Nuclear Factor-kB; p-, phosphorylated; SMAD1, SMAD2, SMAD4, SMAD5, Small Mother Against Decapentaplegic homolog 1/2/4/5; TAK-1, TGFβ-activated kinase 1; ZAP70, Tyrosine protein kinase ZAP70. *pos*, positive control spots; *neg*, negative control spots; IC, internal control.

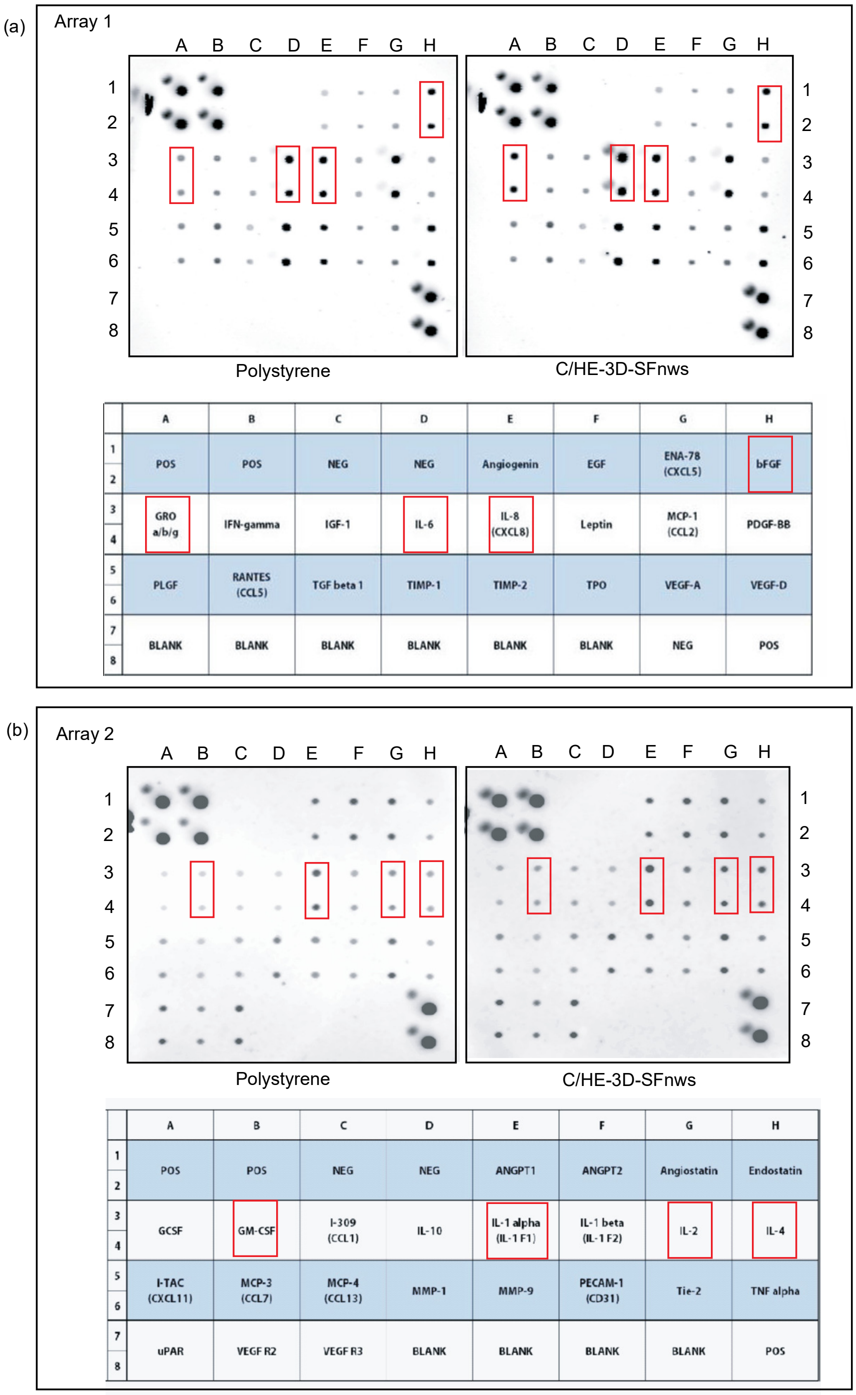

Figure S2. Typical developed double-antibody array membranes (**a**, **b**) showing the angiogenic and trophic factors (AGFs) carried by the exosomes released from AHSMCs grown either as conventional 2D-monolayers on polystyrene (*left*) or on C/HE-3D-SFnws (*right*). Equal amounts of exosomes isolated from AHSMCs -conditioned media samples of the two experimental groups were used. For technical details consult the *Materials & Methods*. The *red rectangles* include the duplicate dots of each of the AGFs whose exosomal amounts were significantly ($P < 0.05$) higher, when comparing the C/HD-3D-SFnws group with the polystyrene group.

Abbreviations, (**a**) EGF, Epidermal growth factor; ENA-78 (CXCL-5), C-X-C motif chemokine ligand 5; bFGF, basic fibroblast growth factor; GRO, growth-regulated oncogene-α/-β/-γ; IFN-γ, Interferon- γ; Interleukin (IL)-1α/-2/-4/-6/-8/-10; monocyte chemoattractant protein (MCP)-1/-2/-3; PDGF-BB, Platelet-Derived Growth Factor BB; PLGF, Placental

Growth Factor; RANTES. (CCL5), Regulated upon Activation, Normal T Cell Expressed and Presumably Secreted; TGF-β, transforming growth factor-beta; TIMP-1/-2, tissue inhibitor of metalloproteinases-1/-2; TPO, thrombopoietin; VEGF-A/-D, Vascular Endothelial Growth Factor-A/-D. (**b**) ANGPT-1/-2, angiopoietin-1/-2; GCSF, granulocyte colony stimulating factor; GM-CSF, granulocyte-macrophage colony-stimulating factor; I-309 (CCL1), C-C Motif Chemokine Ligand 1; I-TAC (CXCL11), C-X-C motif chemokine ligand 11; MMP-1/-9, matrix metalloprotease-1/-9; PECAM-1, Platelet and Endothelial Cell Adhesion Molecule-1; Tie-2, angiopoietin-1 receptor; TNF-α, tumor necrosis factor-α; uPAR, urokinase-like plasminogen activator surface receptor; VEGFR-2/-3, Vascular endothelial growth factor receptor-2/3; *pos*, positive control spots; *neg*, negative control spots.

Relevant trophic and angiogenic actions of AGFs conveyed in increased amounts by exosomes released from C/HE-3D-SFnws-stuck AHSMCs.

In the following paragraphs we will sum up the main biological and applicative features of each the enriched AGFs within the exosomes released from 3D-SFnws-adhering AHSMCs.

IL-1

IL-1α and IL-1β belong to the IL-1 cytokine family, whose signalling regulates angiogenesis and vascular permeability [1]. IL-1α promotes angiogenesis *in vivo* by inducing VEGF's synthesis and secretion by inflammatory cells, and by activating the VEGF•VEGFR-2 signalling pathway between inflammatory cells and blood vessels ECs. Factors like TGF-β, hypoxia [2], and TNF-α [3] upregulate IL-1 in HECs and vascular SMCs. In addition, IL-1 stimulates PDGF's A chain [4], and bFGF expression [5]. Remarkably, IL-1α can also induce its own expression in vascular SMCs [6], thereby exerting autocrine growth-stimulatory effects on the latter [4,6].

IL-2

IL-2 is a 15-kDa glycoprotein first identified as a T cell autocrine growth factor since T cells are the main IL-2 producers. Subsequent reports showed that also ECs [7] and SMCs [8] express IL-2. In the human large and small blood vessels, IL-2 acts on both ECs and SMCs. It directly affects the permeability of ECs [9] and promotes ECs angiogenesis through the α and β IL-2 receptors [10]. As well, by activating Akt/PKB and increasing ROS production, IL-2 stimulated angiogenesis in an animal model and tube formation in human umbilical vein ECs (HUVECs) [10]. In vascular SMCs IL-2 stimulated GAG synthesis and enhanced SMCs responsiveness to angiotensin II [11]. Finally, by playing an autocrine role in cooperation with IL-1α, IL-2 can affect and potentiate human SMCs proliferation [12].

IL-4

IL-4 is an anti-inflammatory cytokine hindering the production and secretion of proinflammatory cytokines, chemokines, proteases, and ROS [13]. IL-4 increases the expression of vascular cell adhesion molecule (VCAM)-1, IL-6, and monocyte chemotactic protein (MCP)-1 [14] in HUVECs. It also induces cytoskeletal rearrangements both in HUVEC and in human coronary artery ECs through a Wnt-5A-mediated stabilization of filamentous (F)-actin cytoskeleton involving a LIM kinase-mediated phosphorylation and inactivation of Cofilin family members [15]. Furthermore, IL-4 acts as a mild mitogen for both macro- and microvascular ECs [16-18].

IL-6

IL-6 stands out as the most intensely enriched cytokine transported by the exosomes released from C/HE-3D-SFnws-supported AHSMCs. IL-6 is an established biomarker of cell survival, growth, and differentiation with pleiotropic pro-inflammatory and anti-inflammatory functions [19]. Vascular SMCs, fibroblasts, monocytes, epithelial cells, glial cells, and cardiomyocytes synthesize IL-6. Pro-inflammatory factors like IL-1, TNF-α, MCP-1, and other pro-inflammatory stimuli can bring about IL-6 expression in vascular SMCs [20]. IL-6 exerts autocrine growth-stimulating

effects on vascular SMCs by inducing the latter to produce endogenous PDGF [21]. Human vascular SMCs express both the membrane-bound IL-6 receptor (IL-6-RA) and the soluble glycoprotein-130 trans receptor (IL6ST), both sources of signals stimulating the autocrine growth and angiogenic activity of ECs [22,23]. Exogenously added IL-6 promotes vascular SMCs proliferation and migration and stimulates their production of ECM-remodelling MMP-1 and MMP-9 [24]. The exposure of vascular ECs to exogenously added IL-6 exerted a weak effect on their migration, proliferation, or tube formation activities, while the autocrine activation by endogenous IL-6 of its classic-signalling pathway was vital to support ECs' cell proliferation and mobility [25].

IL-8/CXCL8, GRO-α/CXCL1, GRO-β/CXCL2 and GRO-γ/CXCL3

Chemokines are a superfamily of homologous 8-10 kDa heparin-binding cytokine molecules mediating leukocyte recruitment at inflammation sites. Chemokine ligands and receptors function as critical intermediaries of neovascularization in diverse physiologic and pathologic settings. They also take part in the pathogenesis of chronic inflammation, fibroproliferative disorders, malignancy, and wound repair [26]. Among the CXC chemokine family, those having the evolutionary 'ELR' motif (i.e. the Glu-Leu-Arg sequence) such as IL-8/CXCL8, GRO-α/CXCL1, GRO-β/CXCL2 and GRO-γ/CXCL3, are all powerful *promoters* of angiogenesis [26-28]. By interacting with the CXCR2 receptor they induce ECs to proliferate, migrate, and form tubes, advancing neovascularization even in the absence of any concurrent tissue inflammation [28]. Under basal conditions vascular SMCs produce extremely low amounts of IL-8/CXCL8; however, agents such as TNF-α, epidermal growth factor (EGF) and IL-1 may increase its expression [26].

bFGF

SMCs do express bFGF, which is a potent mitogen for diverse cell types, and plays a prominent role in the proliferative response to vascular injury. bFGF regulates both angiogenesis and arteriogenesis (reviewed in [29]) and enhances ECs and SMCs proliferation [30]. bFGF also acts as a survival factor for an assortment of quiescent or terminally differentiated cells. Notoriously, autocrine mechanisms mediate both cell survival and cell proliferation. A study about the early postnatal regulation of rat coronary angiogenesis proved that (*i*) both bFGF and VEGF modulate capillary growth; (*ii*) bFGF eases arteriolar growth; and (*iii*) interactions between bFGF and VEGF help set up the normal hierarchy of arteriolar trees [30]. In addition, bFGF plays an important function in ECs too: it may affect angiogenesis by controlling the nuclear translocation and transcriptional activity of β-catenin. In fact, β-catenin•TCF/LEF (T-Cell Factor/Lymphoid Enhancer Factor) transcriptional activity regulates vascular remodelling and the proliferation of human dermal microvascular ECs [31,32].

GM-CSF (Granulocyte Macrophage-Colony Stimulating Factor)

AHSMCs constitutively express GM-CSF and do it more intensely when in the proliferative/secretory phenotype [33]. In addition, GM-CSF can induce and support the proliferation of ECs leading to the formation of endothelial capillaries [34]. GM-CSF also stimulates the migratory repair of mechanically wounded ECs monolayers [35]. Studies using human and animal models proved the ability of GM-CSF to stimulate angiogenesis and neovascularization, arteriogenesis included [36]. However, compared to bFGF, GM-CSF promoted lower peak levels of ECs proliferation, while stimulated ECs migration with the same intensity [35].

References

1. Fahey, E.; Doyle, S. L. IL-1 Family Cytokine Regulation of Vascular Permeability and Angiogenesis. *Front. Immunol.* **2019**, *10*, 1426, doi:10.3389/fimmu.2019.01426

2. Cooper, A.L.; Beasley, D. Hypoxia stimulates proliferation and interleukin-1α production in human vascular smooth muscle cells. *Am. J. Physiol. Heart Circ. Physiol.* **1999**, *277*, H1326-H1337, doi:10.1152/ajpheart.1999.277.4.H1326

3. Libby, P.; Sukhova, G.; Lee, R. T.; Galis, Z. S. Cytokines regulate vascular functions related to stability of the atherosclerotic plaque. *J. Cardiovas. Pharmacol*. **1995**, *25*, S9-12, doi:10.1097/00005344-199500252-00003

4. Raines, E. W.; Dower, S. K.; Ross, R. Interleukin-1 mitogenic activity for fibroblasts and smooth muscle cells is due to PDGF-AA. *Science* **1989**, *243*, 393-396, doi:10.1126/science.2783498

5. Gay, C. G.; Winkles, J. A. Interleukin 1 regulates heparin-binding growth factor 2 gene expression in vascular smooth muscle cells. *Proc. Natl. Acad. Sci. U S A*,**1991**, *88*, 296-300, doi:10.1073/pnas.88.1.296

6. Beasley, D.; Cooper, A. L. Constitutive expression of interleukin-1α precursor promotes human vascular smooth muscle cell proliferation. *Am. J. Physiol. Heart Circ. Physiol.* **1999**, *276*, H901-H912, doi:10.1152/ajpheart.1999.276.3.H901

7. Duechting, A.; Tschöpe, C.; Kaiser, H.; Lamkemeyer, T.; Tanaka, N.; Aberle, S.; Lang, F.; Torresi, J.; Kandolf, R.; Bock, C. T. Human parvovirus B19 NS1 protein modulates inflammatory signaling by activation of STAT3/PIAS3 in human endothelial cells. *J. Virol.* **2008**, *82*, 7942–7952, doi:10.1128/JVI.00891-08

8. Takahashi, Y.; Fujioka, Y.; Takahashi, T.; Domoto, K.; Takahashi, A.; Taniguchi, T.; Ishikawa, Y.; Yokoyama, M. Chylomicron remnants regulate early growth response factor-1 in vascular smooth muscle cells. *Life Sci*. **2005**, *77*, 670-682, doi:10.1016/j.lfs.2005.01.012

9. Downie, G. H.; Ryan, U. S.; Hayes, B. A.; Friedman, M. Interleukin-2 directly increases albumin permeability of bovine and human vascular endothelium in vitro. *Am. J. Respir. Cell Mol. Biol.* **1992**, *7*, 58–65, doi:10.1165/ajrcmb/7.1.58

10. Bae, J.; Park, D.; Lee, Y. S.; Young, D. Interleukin-2 promotes angiogenesis by activation of Akt and increase of ROS. *J. Microbiol. Biotechnol*. **2008**, 18, 377–382.

11. Nabata, T.; Fukuo, K.; Morimoto, S.; Kitano, S.; Momose, N.; Hirotani, A.; Nakahashi, T.; Nishibe, A.; Hata, S.; Niinobu, T.; et al. Interleukin-2 modulates the responsiveness to angiotensin II in cultured vascular smooth muscle cells. *Atherosclerosis* **1997**, *133*, 23–30, doi:10.1016/s0021-9150(97)00107-x

12. Schultz, K.; Murthy, V.; Tatro, J. B.; Beasley, D. Endogenous interleukin-1 alpha promotes a proliferative and proinflammatory phenotype in human vascular smooth muscle cells. *Am. J. Physiol. Heart Circ. Physiol.* **2007**, *292*, H2927–H2934, doi:10.1152/ajpheart.00700.2006

13. Wills-Karp, M.; Finkelman, F. D. Untangling the complex web of IL-4- and IL-13-mediated signaling pathways. *Sci. Signal*. **2008**, *1*, pe55, doi:10.1126/scisignal.1.51.pe55

14. Lee, Y. W.; Eum, S. Y.; Chen, K. C.; Hennig, B.; Toborek, M. Gene expression profile in interleukin-4-stimulated human vascular endothelial cells. *Mol. Med.* 2004, *10*, 19-27, doi:10.2119/2004-00024.lee

15. Skaria, T.; Burgener, J.; Bachli, E.; Schoedon, G. IL-4 Causes Hyperpermeability of Vascular Endothelial Cells through Wnt5A Signaling. *PLoS One* **2016**, *11*, e0156002, doi:10.1371/journal.pone.0156002

16. Klein, N. J.; Rigley, K. P.; Callard, R. E. IL-4 regulates the morphology, cytoskeleton, and proliferation of human umbilical vein endothelial cells: relationship between vimentin and CD23. *Int. Immunol.* **1993**, *5*, 293, doi:10.1093/intimm/5.3.293

17. Toi, M.; Harris, A. L.; Bicknell, R. Interleukin-4 is a potent mitogen for capillary endothelium. *Biochem. Biophys. Res. Commun*. **1991**, *174*, 1287–1293, doi:10.1016/0006-291x(91)91561-p

18. Fukushi, J.; Morisaki, T.; Shono, T.; Nishie,A.;Torisu, H.;Ono, M.; Kuwano, M. Novel biological functions of interleukin-4: formation of tube-like structures by vascular endothelial cells in vitro and angiogenesis in vivo. *Biochem. Biophys. Res .Commun.* **1998**, *250*, 444-448, doi:10.1006/bbrc.1998.9334

19. Schaper, F.; Rose-John, S. Interleukin-6: Biology, signaling and strategies of blockade. *Cytokine Growth Factor Rev.* **2015**, *26*, 475-487, doi:10.1016/j.cytogfr.2015.07.004

20. Villar-Fincheira, P.; Sanhueza-Olivares, F.; Norambuena-Soto, I.; Cancino-Arenas, N.; Hernandez-Vargas, F.; Troncoso, R.; Gabrielli, L.; Chiong, M. Role of Interleukin-6 in Vascular Health and Disease. *Front. Mol. Biosci*. **2021**, *8*, 641734, doi:10.3389/fmolb.2021.641734

21. Ikeda. U.; Ikeda, M.; Oohara, T.; Oguchi, A.; Kamitani, T.; Tsuruya, Y.; Kano, S. Interleukin 6 stimulates growth of vascular smooth muscle cells in a PDGF-dependent manner. *Am. J. Physiol.* **1991**, *260*, H1713–H1717, doi:10.1152/ajpheart.1991.260.5.H1713
22. Klouche, M.; Bhakdi, S.; Hemmes, M.; Rose-John, S. Novel path to activation of vascular smooth muscle cells: up-regulation of gp130 creates an autocrine activation loop by IL-6 and its soluble receptor. *J. Immunol.* **1999**, *163*, 4583–4589.
23. Zegeye, M.M.; Andersson, B.; Sirsjö, A.; Ljungberg, L.U. IL-6 trans-Signaling Impairs Sprouting Angiogenesis by Inhibiting Migration, Proliferation and Tube Formation of Human Endothelial Cells. *Cells* **2020**, *9*, 1414, doi:10.3390/cells9061414
24. Zhu, Y.; Hojo, Y.; Ikeda, U.; Takahashi, M.; and Shimada, K. Interaction between monocytes and vascular smooth muscle cells enhances matrix metalloproteinase-1 production. *J. Cardiovasc. Pharmacol.* **2000**, *36*, 152–161, doi:10.1097/00005344-200008000-00003
25. Ljungberg, L.U.; Zegeye, M.M.; Kardeby, C.; Fälker, K.; Repsilber, D.; Sirsjö, A. Global Transcriptional Profiling Reveals Novel Autocrine Functions of Interleukin 6 in Human Vascular Endothelial Cells. *Mediators Inflamm*. **2020**, *2020*, 1–12, doi: 10.1155/2020/4623107
26. Keeley, E. C.; Mehrad, B.; Strieter, R. M. Chemokines as mediators of neovascularization. *Arterioscler. Thromb. Vasc. Biol.* **2008**, *28*, 1928–1936. doi:10.1161/ATVBAHA.108.162925
27. Koch, A. E.; Polverini, P. J.; Kunkel, S. L.; Harlow, L. A.; Di Pietro, L. A.; Elner, V. M, Elner, S. G.; Strieter, R. M. Interleukin-8 as a macrophage-derived mediator of angiogenesis. *Science* **1992**; *258*, 1798–1801, doi:10.1126/science.1281554
28. Strieter, R. M.; Polverini, P. J.; Kunkel, S. L.; Arenberg, D. A.; Burdick, M. D.; Kasper, J.; Dzuiba, J.; Van Damme, J.; Walz, A.; Marriott, D. The functional role of the ELR motif in CXC chemokine-mediated angiogenesis. *J. Biol. Chem*. **1995**, *270*, 27348–27357, doi:10.1074/jbc.270.45.27348
29. Presta, M.; Dell'Era, P.; Mitola, S.; Moroni, E.; Ronca, R.; Rusnati, M. Fibroblast growth factor/fibroblast growth factor receptor system in angiogenesis. *Cytokine Growth Factor Rev.* **2005**, *16*, 159–178, doi:10.1016/j.cytogfr.2005.01.004
30. Tomanek, R.J.; Hansen, H. K.; Christensen, L. P. Temporally expressed PDGF and FGF-2 regulate embryonic coronary artery formation and growth. *Arterioscler. Thromb. Vasc. Biol*. **2008**, *28*, 1237-1243, doi:10.1161/ATVBAHA.108.166454
31. Holnthoner, W.; Pillinger, M.; Groger, M.; Wolff, K.; Ashton, A. W.; Albanese, C.; Neumeister, P.; Pestell, R. G.; Petzelbauer, P. Fibroblast growth factor-2 induces Lef/Tcf-dependent transcription in human endothelial cells. *J. Biol. Chem.* **2002**, *277*, 45847-45853, doi:10.1074/jbc.M209354200
32. Wang, X.; Xiao, Y.; Mou, Y.; Zhao, Y.; Blankesteijn, W. M.; Hall J.L. A role for the beta-catenin/T-cell factor signaling cascade in vascular remodeling. *Circ. Res.,* **2002**, *90*, 340-347, doi:10.1161/hh0302.104466
33. Plenz, G.; Koenig, C.; Severs, N. J.; & Robenek, H. Smooth muscle cells express granulocyte-macrophage colony-stimulating factor in the undiseased and atherosclerotic human coronary artery. *Arterioscler. Thromb. Vasc. Biol.* **1997**, *17*, 2489–2499, doi:10.1161/01.atv.17.11.2489
34. Krubasik, D.; Eisenach, P. A.; Kunz-Schughart, L. A.Granulocyte-macrophage colony stimulating factor induces endothelial capillary formation through induction of membrane-type 1 matrix metalloproteinase expression in vitro. *Int. J. Cancer* **2008**; *122*, 1261–1272, doi:10.1002/ijc.23234
35. Bussolino, F.; Ziche, M.; Wang, J. M.; Alessi, D.; Morbidelli, L.; Cremona, O.; Bosia, A.; Marchisio, P. C.; Mantovani, A. In vitro and in vivo activation of endothelial cells by colony-stimulating factors. *J. Clin. Invest.* **1991**, *87*, 986–995. doi:10.1172/JCI115107
36. Buschmann, I. R.; Busch, H. J.; Mies, G.; Hossmann, K. A. Therapeutic induction of arteriogenesis in hypoperfused rat brain via granulocyte-macrophage colony-stimulating factor. *Circulation*. **2003**, *108*, 610-615, doi:10.1161/01.CIR.0000074209.17561.99
